# Salicylic Acid in the Treatment of Acute Rheumatism

**Published:** 1892-05-21

**Authors:** 


### SALICYLIC ACID IN THE TREATMENT OF ACUTE RHEUMATISM.

The introduction of remedies which rapidly relieve the acuter symptoms of any painful affection, such as acute rheumatism, is prone to lead to a more or less stereotyped mode of treatment. The treatment of acute rheumatism by salicylic acid or the salicylates has been so widely endorsed that we may sometimes be in danger of forgetting some of the defects in their action. Recently, attention has been re-directed to the advantage of employing the natural acid, but Professor Latham in his Croonian Lectures (1886) has already laid down certain conditions which must be observed to ensure success in the administration of the remedy, viz., (1) the true salicylic acid obtained from the vegetable kingdom must alone be employed. If you have to give large doses, avoid the artificial product obtained from carbolic acid, however much it may have been dialysed and purified. An impure acid will very quickly produce symptoms closely resembling delirium tremens. (2) Give the acid without any base. (3) Place the patient fully under the influence of the drug—that is, let him have sufficient to produce cerebral disturbance, *i.e.*, buzzing in the ears or headache, or slight deafness; with the development of these symptoms the temperature and pains in the joints will begin to decline. To an adult he generally administers three doses of twenty grains, at intervals of an hour, and if the head remains unaffected, a further dose at the end of another hour, and then repeats the twenty grains every four hours until the physiological effect of the remedy shows itself. In the majority of cases eighty to one hundred grains are enough.

It would appear that as long as the rheumatic poison is circulating in the system, the physiological effect—that is, the effect it produces in the healthy organism—does not show itself; acting as an antidote, the greater the amount of poison the larger must be the dose of the remedy; but as soon as the formation of the *materies morbi* is stopped, then the excess of the remedy acts as it would in the healthy organism, and its peculiar physiological effects are developed. (4) Give the patient from forty to eighty grains daily for ten days after all pain and pyrexia have passed away. (5) Let the patient's diet consist entirely of milk and farinaceous food for at least a week after the evening temperature has been normal. On the other hand, if the patient has meat and soup, you may look forward with fair probability to a relapse. (6) Take care to maintain a daily and complete action of the bowels. Calomel is the best purgative. (7) Let the patient be enveloped in a light blanket, and with no more bedclothes than are sufficient to keep him from feeling cold. The object of the treatment now is to cool the patient—not, as in former times, to sweat the poison out of him—and the cooler he is kept the sooner will the temperature be lowered. As regards purgation, Dr. Latham says that the benefits resulting generally in rheumatism from the purgative plan of treatment have always been recognised by the older physicians as striking and satisfactory. By the judicious use of cholagogue purgatives we eliminate the bile from the intestines, and so remove from the system a quantity of glycocine, which if re-absorbed would lead to the consequent formation of uric acid. Calomel is unquestionably of service here.

Dr. Latham further remarked that he had given salicylic acid where there had been both aortic and mitral mischief; and he had also given it in rheumatism complicated with pericarditis, and had seen no bad result from it. But if pericarditis, or endocarditis, or pneumonia, or pleurisy have been developed, the remedy is powerless over the mischief which is done; it will neutralise the poison producing the mischief so as to stop its extension, but the inflammatory exudations will undergo their usual changes unabbreviated n their course.

It is quite possible that the salicylic acid treatment may introduce an increased liability to certain complications. Thus Dr. Lauriston Shaw has drawn attention to the occurrence of hæmorrhage in salicylism during the treatment of acute rheumatism. He is of opinion that the hacemorrhage is due to some chemical or physical change in the blood, which make it more readily transude through the capillaries, or else to some secondary change in the water in the vessels themselves.

In addition to epistaxis, he records cases of retinal hæmorrhage, bleeding from the gums and hæmaturia. In all the cases that Dr. Shaw has observed the hæmorrhage has been preceded by the symptoms usually indicative of too large a dose. The practical conclusion is, that when these symptoms occur, either the dose must be decreased or the drug discontinued altogether, so as to prevent the occurrence of a loss of blood which the patient can so ill afford.*

The views of Dr. Haig on the relation of uric acid to certain diabetic diseases have been brought before the notice of the profession so very fully that it is unnecessary to allude to them in detail. He recently read a paper before the Royal Medical and Chirurgical Society† on salicine compared with salicylate of sodium, as to its effect on the excretion of uric and its value in acute rheumatism. He said that salicine was generally considered to be of less value than a salicylate in acute rheumatism, and then cited cases which corroborated this statement. Dr. Haig further remarked that all methods of treatment that were of any value in acute rheumatism caused a plus excretion of uric acid, and salicine and the salicylates were useful in this disease in exact proportion to their power of producing elimination of this substance. He asked if acute rheumatism were due to uric acid; he gave reasons for thinking that the joint symptoms were due to it, and he considered objections against this view. He pointed out the differences between gout and rheumatism, which, he said, were partly due to the difference in the metabolic processes of young and old. Sir A. Garrod said there was an absence of uric acid from the blood in acute rheumatism, and he had suggested that inflammation destroyed uric acid. Dr. Haig believes that the uric acid was all driven out of the blood by high acidity and precipitated in the joints, and that such precipitation and not destruction of uric acid explained Sir A. Garrod's observation with regard to the effect of inflammation and the fever of rheumatism.

But when we come to weigh the advantages of the newer methods of treating acute rheumatism with salicylates against other medicinal remedies, observations such as those Dr. Donald Hood brought before the Medical Society of London, a few years ago, are more valuable than any number of *ex cathedra* statements of opinion on the point. He based his paper on the reports of more than two thousand cases. Of these 850 were from the records of Guy's Hospital, before the introduction of salicylates; the remainder were all treated with salicylates from the time of admission into the hospital, 515 of them being treated at St. Bartholomew's Hospital, and 635 at Guy's. The cases chosen were all typical acute sthenic rheumatism, occurring in patients of both sexes under 35 years of age. Two marked results of administration of salicylates were a loss of joint pain and a fall in the temperature, but relapses were more common than under the older methods of treatment. The salicylate appeared to have no effect whatever in reducing, preventing, or limiting the intensity of cardiac lesions. The formidable complication of hyperpyrexia was discussed, and cases referred to illustrating the characteristic symptoms; it was as common among patients treated with salicylates as among those treated on general principles. It might occur even when the drug had been given regularly in full doses. For treatment of this complication he recommended to stop administration of this drug, to apply cold locally, and to give stimulants. His more recent enquiries confirmed the opinion he expressed in 1881, that patients taking salicylates, though quickly losing their pain, were often left enfeebled, and remained in hospital longer than those who did not have the remedy.

In conclusion, Dr. Hood believed that in cases of ordinary acute sthenic rheumatism salicylates might be given with advantage, and the advantages were greater and more permanent where they were exhibited in combination with alkalies; but when the attack was complicated with the severer forms of pulmonary or cardiac inflammation, under which circumstances there was usually a coincident mitigation, or even absence of joint pain, he did not think there was the same indication for using the drug.*

How salicylic acid and the salicylates act is not known, but it undoubtedly affects very markedly the nerve centres, and, in a sufficient dose, seems to be a paralysant to all the higher nerve centres. (W. S. Ph.) Clinical experience would at any rate lead to the conclusion that although the acuter symptoms may be quickly relieved, the drug must be used with caution, and should on no account be pushed further than is absolutely necessary to relieve the patient.

* *Lancet*, Jan. 17, 1889. † *Med. Ann.*, 1871, p. 427.

* *Med. Ann.*, 1889. 424.

